# Oxidative Stress, Proton Fluxes, and Chloroquine/Hydroxychloroquine Treatment for COVID-19

**DOI:** 10.3390/antiox9090894

**Published:** 2020-09-21

**Authors:** Christina B. Klouda, William L. Stone

**Affiliations:** Department of Pediatrics, East Tennessee State University, Johnson City, TN 37614, USA; klouda@etsu.edu

**Keywords:** oxidative stress, COVID-19, chloroquine, hydroxychloroquine, reactive oxygen species, proton fluxes, SARS-CoV-2

## Abstract

Chloroquine (CQ) and hydroxychloroquine (HCQ) have been proposed as treatments for COVID-19. These drugs have been studied for many decades, primarily in the context of their use as antimalarials, where they induce oxidative stress-killing of the malarial parasite. Less appreciated, however, is evidence showing that CQ/HCQ causes systemic oxidative stress. In vitro and observational data suggest that CQ/HCQ can be repurposed as potential antiviral medications. This review focuses on the potential health concerns of CQ/HCQ induced by oxidative stress, particularly in the hyperinflammatory stage of COVID-19 disease. The pathophysiological role of oxidative stress in acute respiratory distress syndrome (ARDS) has been well-documented. Additional oxidative stress caused by CQ/HCQ during ARDS could be problematic. In vitro data showing that CQ forms a complex with free-heme that promotes lipid peroxidation of phospholipid bilayers are also relevant to COVID-19. Free-heme induced oxidative stress is implicated as a systemic activator of coagulation, which is increasingly recognized as a contributor to COVID-19 morbidity. This review will also provide a brief overview of CQ/HCQ pharmacology with an emphasis on how these drugs alter proton fluxes in subcellular organelles. CQ/HCQ-induced alterations in proton fluxes influence the type and chemical reactivity of reactive oxygen species (ROS).

## 1. Introduction

**Figure 1:**
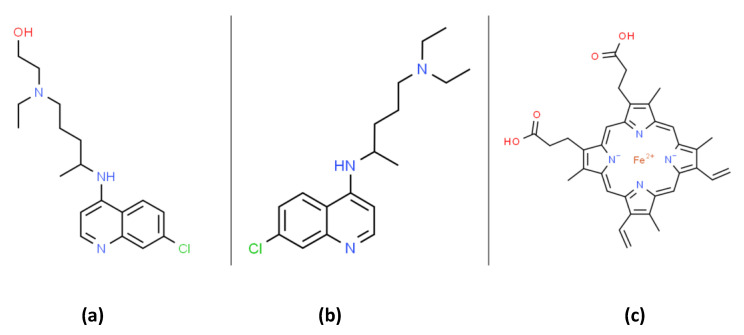
The organic structures of (**a**) hydroxychloroquine (HCQ); (**b**) chloroquine and; (**c**) heme. Chloroquine can form a membrane bound complex with heme that can promote lipid peroxidation (see Figure 4 and text).

At the writing of this review (mid-2020), there was no Food and Drug Administration (FDA) approved drugs for severe acute respiratory syndrome coronavirus 2 (SARS-CoV-2) RNA virus infection, which causes the coronavirus disease 2019 (COVID-19). Both chloroquine (CQ) and hydroxychloroquine (HCQ) are 4-aminoquinoline drugs with similar structures, as shown in Figure 1. CQ and HCQ have been proposed as potential COVID-19 medical countermeasures [1,2,3,4,5,6]. The FDA currently approves CQ/HCQ for use as antimalarials, the treatment of rheumatoid arthritis, and systemic lupus erythematosus (SLE) where they act by immunomodulatory mechanisms [7]. HCQ reduces disease activity in SLE patients but has no significant effect on pro-inflammatory cytokines [8]. A recent literature review highlights many immunomodulatory mechanisms influenced by CQ/HCQ and emphasizes our lack of current knowledge concerning their potential effects on immune responses in COVID-19 patients [9]. CQ/HCQ use may lead to unknown alterations in immune responses in COVID-19 patients, including diminished innate immune responses as well as potential modifications of the B and T cell responses to the COVID-19 virus [9]. 

Both in vitro and in vivo animal experiments have demonstrated the anti-coronavirus activity of CQ /HCQ [10,11,12]. Moreover, CQ/HCQ are broadly available, cost-effective, and therefore attractive potential therapies for viral pandemics without an effective vaccine. A review by Cortegiani et al. on the efficacy and safety of CQ for treating COVID-19 concluded that available pre-clinical evidence was sufficiently robust to justify initiating high-quality clinical trials [4]. Based on available data, the FDA initially published an emergency use authorization of CQ/HCQ (on 28 March 2020) for COVID-19 treatment. The FDA subsequently revoked this emergency use authorization (on 15 June 2020) due to new clinical data suggesting CQ/HCQ were ineffective and raising concerns regarding “serious cardiac adverse events and other potential serious side effects” [13]. Nevertheless, the FDA states “additional clinical trials continue to evaluate the potential benefit of these drugs in treating or preventing COVID-19. “

The molecular basis for the potential antiviral activity of CQ/HCQ are not fully understood, and multiple mechanisms have been proposed [9]. This review will explore the hypothesis that alterations in proton fluxes and redox physiology induced by CQ/HCQ are relevant to their potential antiviral activity and side-effects. The production of reactive oxygen species (ROS) and cellular/subcellular proton fluxes are interdependent factors modulating many antimicrobial immune responses [14]. We will also highlight concerns that CQ/HCQ-induced oxidative stress could be problematic in the treatment of critically ill COVID-19 patients. The “cytokine storm” occurring in some severe forms of viral infection (e.g., influenza A viruses) is associated with increased oxidative stress as well as increased morbidity and mortality [15]. Accumulating evidence suggests that this cytokine storm is a significant cause of ARDS and multiorgan failure in COVID-19 patients [16].

Considerable evidence suggests that parasite-specific oxidative stress induced by CQ/HCQ treatment accounts for the antimalarial activity of these drugs [17]. Less appreciated, however, is evidence (see below) showing that CQ/HCQ, by themselves, are pro-oxidants that can increase oxidative stress parameters [18,19,20,21]. Viral infections are also generally accompanied by oxidative stress with potential pathophysiological consequences [22]. As mentioned above, compelling evidence supports the view that patients with COVID-19 are at increased risk of developing ARDS and subsequent death from respiratory failure [23]. Decades of research have demonstrated the central role of oxidative stress in ARDS pathophysiology [24,25,26,27]. Understanding the potential role of oxidative stress in COVID-19 is critical, since potential anti-COVID-19 drug candidates that increase oxidative stress have the potential to exacerbate ARDS pathophysiology.

## 2. CQ/HCQ Pharmacology and Alterations in Subcellular Organelles Proton Fluxes

**Figure 2:**
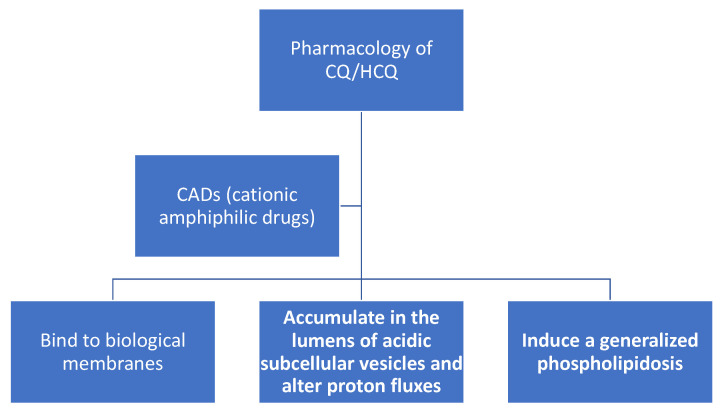
Chloroquine (CQ) and hydroxychloroquine (HCQ) are cationic amphiphilic drugs (CADs) that share a set of common pharmacological properties: (a) they can partition into biomembranes; (b) accumulate in the lumens of acidic subcellular organelles, and; (c) induce a generalized phospholipidosis, which is the lysosomal accumulation of phospholipids.

Before reviewing the role of CQ/HCQ in redox physiology and SARS-CoV-2 infection, we will briefly summarize the basics of CQ/HCQ pharmacology (see Figure 2) with an emphasis on CQ/HCQ-induced alterations in subcellular organelle proton fluxes. CQ/HCQ-induced changes in proton fluxes will have a direct effect on the type of ROS present, and thereby influence their potential effects on redox-sensitive signal transduction pathways and potential molecular and cellular damage. Among the key redox-sensitive pathways are those controlled by transcription factor nuclear factor erythroid 2 p45-related factor 2 (NRF2), hypoxia-inducible transcription factors (HIFs), nuclear factor-κB (NF-κB) transcription factors, and activator protein-1 (AP-1) transcription factors (see below as well). Excellent, comprehensive reviews of CQ/HCQ pharmacokinetics and their side-effects are available [9,28,29].

## 3. CQ/HCQ Bind to Biological Membranes and Alters their Structure/Function

After many decades of research, some of the least controversial characteristics of CQ/HCQ are the ability of these weakly basic and lipophilic compounds: (1) to bind biological membranes; (2) to accumulate in the lumens of acidic subcellular vesicles and alter proton fluxes; (3) to induce generalized cellular phospholipidosis. These effects are relevant to the production of ROS, proton fluxes and the immunomodulatory and potential antiviral activities of CQ/HCQ.

CQ and HCQ both belong to a class of compounds (see Figure 1 and Figure 2) termed cationic amphiphilic drugs or CADs [30]. In plasma (pH 7.4) and cellular cytoplasm (pH 7.2), the divalent forms of CQ/HCQ are the dominant species. At pH 7.4 CQ, CQH^+^ (monovalent), and CQH_2_^2+^ (divalent) are present at 0.026, 16.74, and 83.23%, respectively (reaction 1). Unambiguous in vitro experiments show that CQH_2_^2+^ binds to the phospholipid bilayers of multilamellar liposomes with a robust partition coefficient [31]. Phosphatidylserine (PS) liposomes, with a negative charge, binds CQH_2_^2+^ with a particularly high partition coefficient [31]. PS plays a central role in apoptosis. Proton NMR studies have confirmed the binding of CQH_2_^2+^ to phosphatidylcholine liposomes [32]. As expected, resonance signals from protons on the hydrophobic ring carbons of CADs are affected by association with liposomes [32].

(1) $$\mathrm{CQ}\;\overset{H^{+}}{⇌}\;{\mathrm{CQH}}^{+}\;\overset{H^{+}}{⇌}\;{{\mathrm{CQH}}_{2}}^{2+}$$

The binding of CADs to lipid bilayers is stabilized by nonspecific hydrophobic and ionic interactions [31]. Lipid bilayers are present in all cells and many subcellular organelles. It follows that CADs can accumulate, to some extent, in the cells and membranous organelles of most tissues. CAD accumulation can potentially affect many functions of all biological membranes by altering their structure and functions, e.g., membrane-bound lysosomal phosphatases and hydrolases [33].

## 4. CQ/HCQ Accumulate in the Lumens of Acidic Subcellular Vesicles and Alter Proton Fluxes

It is also well established that CADs accumulate in the lumens of acidic subcellular vesicles. The neutral forms of CADs can freely diffuse through the hydrophobic domain of lipid bilayers. For acidified vesicles, such as endosomes, lysosomes, M2 phagosomes, and pulmonary lysosomal lamellar bodies, the neutral forms of CADs will become charged inside the lumen and will no longer be able to diffuse out [28]. Acidified vesicles utilize a vacuolar proton-pumping ATPase (V-ATPase) to maintain a low pH by pumping protons across the vesicular membrane into the lumen [34]. For lysosomes attempting to maintain a low pH (pH 4.8) in the presence of CQ/HCQ, there will be a gradual luminal accumulation of CQ/HCQ, eventually leveling off at levels as high as 20 mM [28,35]. This process is termed “lysosomal trapping,” and trapped CADs are denoted as “lysosomotropic” [35,36,37]. CQ shows a wide variation in lysosomal trapping between organs with lungs > kidney = brain = liver > diaphragm = heart = skeletal muscles > adipose tissue [38]. The high CQ accumulation in the lungs is relevant to respiratory distress disorders [39].

Lamellar bodies are a type of acidified lysosome found in type II alveolar cells (T2ACs), and keratinocytes and these organelles are known to accumulate weak bases such as CQ/HCQ [40,41]. The lamellar bodies found in T2ACs secrete surfactant, which is essential for pulmonary alveoli gas exchange [42]. T2ACs are particularly relevant to COVID-19, since these cells express angiotensin-converting enzyme-2 (ACE-2), which SARS-CoV-2 utilizes as a receptor to enter the lungs. T2AC cells are preferentially infected by SARS-CoV-2, potentially contributing to a reduced secretion or function of surfactant with a resulting loss of pulmonary compliance [43]. Reduced pulmonary compliance is a typical characteristic of ARDS, but exogenous surfactant treatment has not proven to be therapeutically effective [44,45]. Nevertheless, there is a pharmaceutical interest in testing surfactant therapy in COVID-19 patients. CQ interferes with the processing of surfactant protein C, which is necessary for a fully functional surfactant [46]. Moreover, ROS can inactivate surfactant by structural and functional modifications to surfactant proteins SP-B and SP-C [47].

Initial in vitro data suggested that the pH of lysosomes would increase as a result of CAD trapping, but subsequent, more detailed studies do not support this view [30,35,48,49]. Data in an animal model show, for example, that CQ (40 mg/kg body weight) will transiently increase hepatocyte lysosomal pH from 4.8 to 6.8 for about 2 h, followed by a return to pH 4.8 lasting for at least 10 h [49]. Maintaining an acidic lysosomal pH in the face of CQ accumulation necessitates an increased ATP consumption by V-ATPase and an increased proton flux into the lysosomal lumen.

## 5. Endosomal-Lysosomal Proton Fluxes, CQ/HCQ and SARS-CoV-2

A third well-studied effect caused by CADS (see Figure 2) is a generalized lysosomal accumulation of phospholipids termed phospholipidosis. Phospholipidosis is a lysosomal storage disorder characterized by an abnormal accumulation of phospholipids in the form of lamellar bodies [30]. Drugs causing phospholipidosis are recognized as being potentially toxic by the pharmaceutical industry [50,51]. The underlying mechanism(s) for phospholipidosis remains a matter of some controversy. Most likely, CAD inhibition of lysosomal lipid degradation enzymes is involved [30]. Studies in an animal model indicate that CADs can induce pulmonary lesions characterized by large foamy macrophages in the alveolar spaces [51]. The foamy alveolar macrophages show a typical CAD-induced lysosomal phospholipidosis with the potential to interfere with the phagocytosis and catabolism of pulmonary surfactant [51]. The potential pathophysiological consequences of phospholipidosis remain an area of active interest.

The view that lysosomes are relatively inert organelles with a narrow degradative function is rapidly changing [36]. We now appreciate that lysosomes are dynamic organelles playing a central role in a wide variety of signaling pathways affecting immune responses, viral infectivity, the inactivation of microbes, and inflammation [35,52,53]. Alterations in luminal proton fluxes can be induced by both CQ/HCQ and ROS (see below). Proton fluxes can, in turn, influence both the levels and types of ROS [54].

Lysosomes can fuse with endosomes, thereby delivering the endocytosed cargo to lysosomes [55]. Pioneering work by Burkard et al. shows that coronavirus entry into cells can occur via this endolysosomal (or endocytic) pathway [56]. The endolysosomal pathway is under intense scrutiny due to its role as a target for COVID-19 therapy [57]. The spike glycoprotein (S) of SARS-CoV-2 is proteolytically cleaved by cellular serine protease TMPRSS2 into two subunits, S1 and S2 [58]. The S1 viral protein binds to the host cell angiotensin-converting enzyme 2 (ACE2) plasma membrane protein. The SARS-CoV-2 interaction with ACE2 initiates the formation of clathrin-coated pits, which serve as SARS-CoV-2 entry receptors. The virus is then brought into the cell’s cytoplasm via endocytosis with the formation of early endosomes. The early endosomes with SARS-CoV-2 subsequently form late endosomes that fuse with lysosomes. The S2 protein subunit promotes the fusion of the viral membrane with cellular membranes [57,58,59]. Encouragingly, sera from recovered SARS patients can block SARS-CoV-2 host cell entry in a cell culture model [58].

The notion that CQ/HCQ could alkalinize endosomal-lysosomal pH and thereby inhibit the replication of viruses requiring an acidic pH has been an attractive hypothesis [60]. This potential mechanism is often proposed as a rationale for the CQ/HCQ treatment of COVID-19 [61]. Nevertheless, the references cited above cast doubt on this hypothesis, since CQ/HCQ endosomal-lysosomal alkalinization appears to be only transient, lasting only a few hours, but long enough to confound short-term in vitro experiments.

As mentioned above, CQ/HCQ are likely to increase cellular ATP consumption and increase proton flux across the lysosomal membrane. Under conditions where ATP production is decreased (e.g., mitochondrial uncoupling or hypoxia) sufficiently to inhibit V-ATPase activity, it might be possible for CQ/HCQ to induce some degree of endosomal-lysosomal alkalinization [62]. Low V-ATPase protein expression could also cause CQ/HCQ alkalinization. This avenue of research is not well studied. Intracellular ATP levels are also a key determinant governing the mode of cell death: lack of ATP favors necrosis over apoptosis [63]. Although beyond the scope of this review, it should be noted that many viruses have evolved molecular mechanisms to modulate modes of cell death to their advantage [64]. Blocking apoptosis and the subsequent killing of virally infected cells is one such mechanism [64].

## 6. Redox Physiology, Reactive Oxygen Species (ROS) and Oxidative Stress

We will next provide a brief overview of redox physiology and its interconnections to proton fluxes and CQ/HCQ. Redox physiology is an inclusive term referring to the complex role that oxidation-reduction reactions play in both normal and pathophysiological processes, including the role of ROS in modulating signal transduction pathways (e.g., NF-kappaB transcription factor) and inflammatory responses [65]. ROS is a term used to describe small oxygen-containing molecules that are reactive or give rise to reactive species. The name “reactive oxygen species” is preferable to “free radical species”, since not all free radical oxygen species are very reactive, and some non-free radical oxygen species are very reactive, e.g., singlet oxygen. Moreover, hydrogen peroxide is considered a primary ROS and is not a free radical. The primary ROS are superoxide (O_2_^•−^ where • is an unpaired electron), hydrogen peroxide (H_2_O_2_), and the hydroxyl radical (•OH) [66]. Secondary ROS, such as hypochlorous acid (HOCl) and lipid peroxyl radicals (LOO•), can be derived from primary ROS (see below). Oxidative stress is a redox imbalance occurring when the production of ROS and/or reactive nitrogen oxide species (RNOS) is sufficient to cause physiological damage [67]. An overproduction of ROS/RNOS and/or deficient levels of antioxidants (e.g., glutathione) can result in oxidative stress.

## 7. Mitochondria, ROS, Hypoxia, and CQ/HCQ

**Figure 3:**
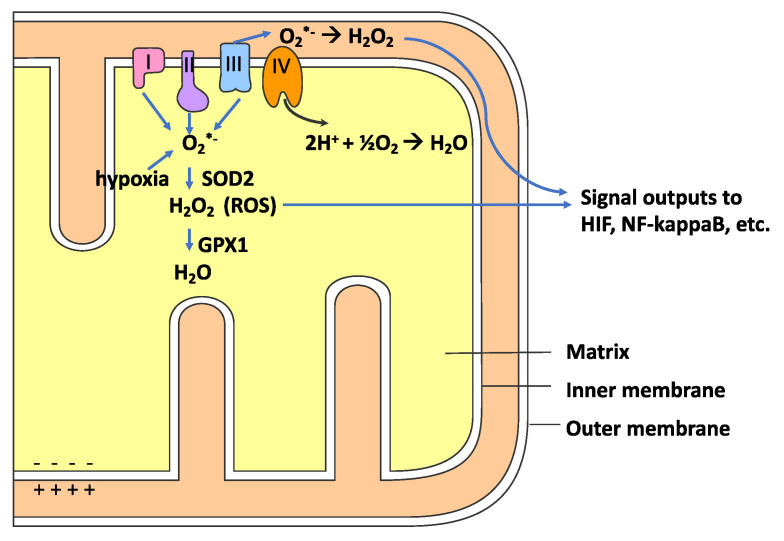
The mitochondrial electron transport chain (ETC) is a major source of reactive oxygen species (ROS). The superoxide anion (O_2_^•−^) is produced when an electron (e^−^) is transferred to oxygen rather than the next electron carrier in the ETC. Complexes I and II produce O_2_^•−^ only in the mitochondrial matrix, whereas complex III can also produce O_2_^•−^ in the intermembrane space.). Complex IV (cytochrome c oxidase) transfers electrons to O_2_ generating H_2_O. Superoxide dismutase 2 (SOD2) reduces O_2_^•−^ to H_2_O_2_. H_2_O_2_ is reduced by glutathione peroxidase 1 (GPX1). Mitochondrial ROS levels modulate many redox-sensitive signal transduction pathways through transcription factors such as nuclear factor kappa B (NF-kappaB) and hypoxia-inducible factor (HIF).

As indicated in Figure 3, the mitochondrial respiratory chain electron transport chain (ETC) is a major source of ROS for most cells undergoing normal metabolism, e.g., noninflammatory conditions [68,69]. The O_2_^•−^ anion is produced when an electron (e−) is transferred to oxygen rather than the next electron carrier in the ETC, as shown in reaction 2. Mitochondrial complexes I and II produce O_2_^•−^ only in the mitochondrial matrix space, whereas complex III can also produce O_2_^•−^ in the intermembrane space (Figure 3) [70].

O_2_^•^ + e^−^ → O_2_^•−^ (2)

HO_2_^•^ ⇌ O_2_^•−^ + H^+^ (pKa = 4.8) (3)

(4) $$2{O_{2}}^{•-}\;+\;2H^{+}\;\overset{\mathrm{SOD}}{\to }\;H_{2}O_{2}\;+\;O_{2}$$

Typically, the low levels of ROS produced by the mitochondria are not enough to cause oxidative stress. Moreover, low ROS levels are increasingly recognized as playing vital roles in modulating many redox-sensitive signal transduction pathways, with the NF-kappaB pathway being particularly relevant to COVID-19 due to its central role in inflammation [71]. The activation of transcription factor NF-kappaB by ROS can induce the expression of pro-inflammatory cytokines and chemokines. NF-kappaB activation also promotes the expression of key antioxidant enzymes such as SOD2, SOD1, HO-1, and GPX [72]. In vitro experiments have demonstrated that CQ is capable of evoking NF-kappaB activation and subsequent expression of pro-inflammatory cytokines in some cell lines [73,74].

Under some conditions, more robust levels of ROS can be produced by the ETC, resulting in oxidative stress and mitochondrial damage [75,76]. As reviewed by Hamanaka et al., hypoxia is a condition that promotes increased mitochondrial ROS production [71]. Hypoxia in COVID-19 patients is associated with clinical deterioration [77].

At pH 7.4, O_2_^•−^ is present as an anion but, at low pH, is protonated to form the hydroperoxyl radical (HO_2_^•^), as indicated in reaction 3. HO_2_^•^ is much more reactive oxygen species than O_2_^•−^. Since HO_2_^•^ is not charged, it can enter lipid bilayers and initiate lipid peroxidation of biological membranes [78]. As discussed below, the pH of an acidified phagosome is typically 4.5, and most of the O_2_^•−^ would be present as HO_2_^•^. Since CQ/HCQ can alter proton fluxes in many subcellular organelles, these drugs can change the type of ROS present and the ability of ROS to damage cells and subcellular organelles.

Mitochondria contain a superoxide dismutase (SOD) enzyme with a manganese cofactor (MnSOD or SOD2) which can reduce O_2_^•−^ to H_2_O_2,_ as indicated in reaction 4. The H_2_O_2_ produced in the mitochondrial matrix can be reduced by selenocysteine-containing glutathione peroxidase (GPX1), which utilizes reduced glutathione (gamma-L-glutamyl-L-cysteinyl glycine or GSH) (see reaction 5). GSH is the major intracellular chemical antioxidant. GPX1 is found in the cytoplasm and mitochondria. As detailed below, CQ induces a systemic decrease in GSH levels. GPX1 can modulate mitochondrial functions that, in turn, can regulate many redox-dependent cellular responses [79]. H_2_O_2_ produced in the mitochondrial matrix can diffuse through the inner and outer mitochondrial membranes to the cytoplasm (see Figure 3).

H_2_O_2_, in the presence of ferrous ions, can be reduced to the highly reactive hydroxyl radical (•OH) which can abstract a hydrogen from a polyunsaturated fatty acid (PUFA) moiety (LH) to form a lipid hydroperoxide (LOOH), as shown in Reactions 6-8. As detailed more below, ferritin functions as an antioxidant protein by preventing redox-active iron ions from generating ROS. The various forms of vitamin E (mostly RRR-alpha-tocopherol and RRR-gamma-tocopherol) can quench the free radicals produced during lipid peroxidation. If not quenched, the LOO• from Reaction 9 can start another cycle of lipid peroxidation (Reactions 7 and 8) since the LH moieties in biomembranes are in close and fluid contact. Vitamin E (TOH) is the major lipid-soluble antioxidant and is present in all biomembranes.

(5) $$H_{2}O_{2}+\;2\mathrm{GSH}\;\overset{\mathrm{GPX}}{\to }\;2H_{2}O\;+\;\mathrm{GSSG}$$

H_2_O_2_ + Fe^2+^ → OH^−^ + ^•^OH + Fe^3+^ (6)

LH + ^•^OH → L^•^ + H_2_O (7)

L^•^ + O_2_ → LOO^•^ (8)

LOO^•^ + LH → LOOH + L^•^ (9)

LOO^•^ + TOH → LOOH + TO^•^ (10)

Only limited data exist on how CQ/HCQ affects mitochondria ROS production or mitochondrial functions in general. As mentioned above, both CQ and HCQ (see Figure 1) are CADs, and the biochemistry of this group of compounds has been intensively studied [31]. Since CADs are positively charged, they can accumulate in the negatively charged mitochondrial matrix, where they could alter proton fluxes, ROS production, and potentially disrupt mitochondrial functions [75].

Although there are minimal data on the potential accumulation of CQ/HCQ in mitochondria, there is clear evidence suggesting that CQ/HCQ affect mitochondrial functions. Early work with rat liver mitochondria shows that CQ alters mitochondrial lipid composition, decreases the activities of NADH dehydrogenase, succinate dehydrogenase, and cytochrome c, and inhibits both mitochondrial respiration and ATP synthesis [80]. Subsequent work confirms that CQ adversely affects rat liver mitochondria by markedly decreasing respiration rates and by acting as an uncoupler. Uncoupling occurs when energy is expended by creating a proton gradient (or protonmotive force) but not utilized in making ATP [81]. In the above-cited work, CQ was administered to rats for extended periods (7 days, 14 days) before isolating mitochondria and characterizing functional alterations. Since CQ is transformed into excretable metabolites by microsomal cytochromes, these metabolites may cause or contribute to the observed mitochondrial changes. In any event, mitochondrial uncoupling is closely linked to ROS production [82]. Moreover, the isolated mitochondria may have been the target of oxidative stress generated elsewhere in the cell rather than the source of oxidative damage.

## 8. Phagocytes, Proton Flux, ROS and CQ/HCQ

Both neutrophils and macrophages play critical roles in the innate immune response by direct respiratory burst ROS-mediated virus killing and by the phagocytosis of apoptotic virus-infected cells [83,84]. These cell types are also consequential in ARDS pathology, which accounts for many of the deaths associated with COVID-19 [23,24]. Phagocytic cells are also central to the cytokine storm, which is considered “the most dangerous and potentially life-threatening event related to COVID-19” [85]. The ROS produced by phagocytic cells can also damage vascular endothelial cells, which is increasingly recognized as significant in the adverse cardiovascular effects of COVID-19 [86,87,88].

The large amount of O_2_^*−^ produced during the “respiratory burst” is accomplished by the transfer of an electron (e−) from nicotinamide adenine dinucleotide phosphate (NADPH) to oxygen (see Reaction 11), as catalyzed by NADPH oxidase (NOX2) [89,90].

(11) $$2O_{2}\;+\;\mathrm{NADPH}\;\overset{\mathrm{NOX}2}{\to }\;2{O_{2}}^{•-}\;+\;{\mathrm{NADP}}^{+}\;+\;H^{+}$$

The O_2_^•−^ produced during the respiratory burst of phagocytic cells is secreted into the phagosomes that have engulfed pathogens [91,92]. The ROS that kills viral and bacterial pathogens is primarily hypochlorous acid (HOCl) produced by myeloperoxidase (MPO), as shown in Reaction 12.

(12) $$H_{2}O_{2}\;+\;{\mathrm{Cl}}^{-}\;\overset{\mathrm{MPO}}{\to }\;\mathrm{HOCl}$$

CQ is a potent in vitro inhibitor of MPO, suggesting that it could compromise the neutrophil killing of microbes [93]. In vitro studies of human polymorphonuclear neutrophils (PMNs) also show that CQ induces dysfunction in chemotaxis, phagocytosis, and respiratory burst [94]. Whether or not these in vitro studies have physiological significance is not clear.

The luminal pH of phagosomes can influence their ability to inactivate microbes [14,52,53]. CQ/HCQ can modulate the pH of phagosomes and thereby modulate the immune responses of macrophages [95]. The O_2_^•−^ generated by reaction 11 occurs in the phagosomal lumen, but the H^+^ generated is released to the cytoplasm [53]. Moreover, the subsequent dismutation of O_2_^•−^ (reaction 4) in the phagosomal lumen will consume H^+^. In the absence of V-ATPase, these events would promote cytosol acidification and phagosome alkalization. The very high levels of O_2_^•−^ produced during the respiratory burst produces major alterations in the proton fluxes in both the macrophage cytosol and phagosomes [53].

Macrophages have phenotypic plasticity called “macrophage polarization,” which enables them to modulate their immunological responses to pathogens and tissue damage. M1 and M2 macrophages represent two extremes of this phenotypic plasticity. M1 macrophages are optimized to kill pathogens, and M2 macrophages are optimized for tissue healing [96]. Proton fluxes in the phagosomes of M1 and M2 are different and differentially modulated by CQ/HCQ. M2 macrophages have a very active V-ATPase and, therefore, can maintain an acidified phagosome after initiation of a respiratory burst [14]. A low phagosomal pH is essential for maintaining the activity of lytic enzymes needed for the degradation of apoptotic cells (as well as virally infected apoptotic cells). In contrast, M1 macrophages have phagosomes with much less V-ATPase and hence will not acidify after the initiation of a respiratory burst [14].

In vitro data indicate that M1 macrophages can generate higher levels of NOX2-generated ROS than M2 macrophages, suggesting that M1 macrophages would be superior to M2 macrophages at killing phagocytized viral pathogens [14]. In contrast, M2 macrophages would be more efficient at digesting dying cells than M1 macrophages, since an acidic pH is optimal for the lytic activity of many phagosomal digesting enzymes [14]. M2 macrophages would be critical for the removal of virally infected apoptotic or necrotic host cells and antigen presentation. Moreover, the phagocytic removal of dying host cells is vital for resolving the inflammation associated with infection and for restoring damaged tissue [97]. M1 macrophages are considered pro-inflammatory and promote tissue damage [95]. In vitro studies by Chen et al. [95] show CQ can “reset” macrophages towards the M1 phenotype due to CQ-induced alkalization of the phagosomal lumen. If this CQ-induced M1-macrophage reset occurred in vivo, it would not bode well for late-stage CQ/HCQ COVID-19 treatment.

Labro et al. studied the in vitro effects of CQ on human polymorphonuclear neutrophil (PMNs) [94]. CQ at 100 µg/mL (but not lower) inhibited both phagocytosis (50%) and the respiratory burst (80%). The CQ concentrations used in these experiments are much higher than typically obtained by oral CQ administration, where a 600 mg dose results in a peak plasma CQ concentration of about 0.3–0.4 µg/mL [94].

## 9. Evidence for Oxidative Stress Induced by CQ/HCQ

We will next review both the in vitro and in vivo evidence for CQ/HCQ-induced oxidative stress. This research is relevant to COVID-19, since accumulating evidence suggests that oxidative stress is linked to the pathology of SARS-CoVs infections [98]. Additional oxidative stress induced by CQ/HCQ could exasperate viral-induced ARDS pathology.

The historical impetus for studying the relationship between CQ/HCQ and oxidative stress relationship springs from the widely held hypothesis that CQ exerts its antimalarial effect by oxidative stress-killing of *Plasmodium falciparum* parasites during the intra-RBC phase of their life cycle [21,99]. Moreover, high doses of CQ are associated with oxidative stress-induced retinopathy [100,101].

## 10. CQ/HCQ Influence on Oxidative Stress In Vivo and Ex Vivo

There are very few clinical studies looking at the connections between CQ/HCQ treatment and oxidative stress. Farombi et al. measured oxidative stress parameters in subjects being treated with CQ for malaria (*Plasmodium falciparum*) [19]. Four groups were studied (*n* = 10 per group): (1) a CQ-treated group with malaria; (2) a CQ-treated group without malaria; (3) a control group not treated and without malaria; (4) a second control group not treated with CQ but having malaria. CQ was provided for three days at a dose of 25 mg/kg body weight. The CQ-treated groups had significantly lower RBC levels of catalase (CAT) and GPX activities compared with the nontreated groups, while SOD1 increased. SOD1 is a copper/zinc form of SOD. The increased RBC SOD1 activity was viewed as an adaptive response to oxidative stress, which is a typical result of such stress. Plasma levels of vitamin A, C, GSH, and beta-carotene (all chemical antioxidants) were significantly decreased by CQ treatment, while malondialdehyde (MDA) levels (a measure of lipid peroxidation) were increased. Low GSH is typically the result of increased GSH consumption by GPX (reaction 5). Vitamin E levels were not measured. Farombi et al. concluded that CQ-treatment-induced systemic oxidative stress in human subjects [19].

Animal models also support the role of CQ treatment in inducing systemic oxidative stress. Ogunbayo [102] examined oxidative stress parameters in the whole blood and serum from rabbits after a single dose (10 mg/kg body weight) of CQ phosphate. Serum GSH levels were found to significantly decrease 6 and 12 h after CQ administration and return to baseline after 24 h. MDA levels were significantly increased 6 h after CQ administration. A decreased GSH serum level and an increased serum MDA level is good evidence for systemic CQ-induced oxidative stress. The RBC activity of two key antioxidant enzymes was also measured in this study. RBC SOD1 significantly increased 6 h after oral CQ administration followed by a return to baseline after 24 h. The increase in SOD1 after 6 h of CQ administration was interpreted as a protective response to increased RBC production of O_2_^•−^. RBC CAT levels were found to be significantly decreased at 6 h. CAT converts H_2_O_2_ to H_2_O, and O_2_^*−^ inactivates its activity. The activity of GPX was not measured in this study, nor were antioxidant enzyme protein levels accessed by Western blots.

A more extensive study examined the effects of long-term CQ and primaquine (PQ) administration on oxidative stress parameters in a rat model [103]. CQ increased kidney MDA levels after 7 and 14 days of CQ administration but not at 21 days. CQ did not alter liver levels of MDA at any time point. Protein carbonyl levels in the kidney were increased by CQ on day 14 but not on day 7 or 21. DNA damage was evaluated using the comet assay, which is considered an indirect measure of DNA strand breaks caused by systemic oxidative stress [104]. DNA damage was markedly increased in the kidney by CQ on day 7 and 14 but not at day 21. Both the brain and liver showed marked increases in CQ induced DNA damage at days 14 and 21.

Bhattacharyya et al. [100] looked at the effects of CQ on NADPH-induced lipid peroxidation, antioxidant enzymes, and the GSH content of the rat retina after both acute and chronic CQ administration. The retina is particularly sensitive to oxidative stress damage since it has the highest polyunsaturated fatty acid (PUFA) content of any tissue as well as the highest oxygen consumption [105]. A high PUFA content in a tissue markedly increases its susceptible to lipid peroxidation [106]. Bhattacharyya et al. [100] found that acute CQ (0.5 and 4 h after intraperitoneal injection) increased NADPH-induced lipid peroxidation and decreased retinal tissue GSH content. In marked contrast, chronic CQ did not induce NADPH-induced lipid peroxidation and increased the retinal GSH content. SOD and GPX activity decreased after both acute and daily CQ while SOD increased only in the high-dose acute study.

While the clinical and animal model evidence reviewed above suggests that CQ can induce systemic oxidative stress, this does not preclude the possibility that CQ/HCQ could simultaneously exert a localized anti-oxidative role. Similarly, the data for CQ-induced systemic oxidative stress do not exclude the possibility of very intense but localized oxidative stress. In an ex vivo experiment, Jancinova et al. found that HCQ (40 mg/kg daily for 21 days) markedly reduced phorbol 12-myristate 13-acetate (PMA)-stimulated oxidant formation in the blood of rats with adjuvant arthritis [107]. Human neutrophils were also examined, and HCQ was found to decrease PMA-stimulated extracellular neutrophil oxidants but to increase intracellular oxidant formation. The physiological significance of these results to CQ/HCQ treatment of COVID-19 is not clear but suggests that under some circumstances, HCQ can act to prevent ROS- induced inflammation.

## 11. CQ/HCQ Oxidative Stress In Vitro and the Role of Free-Heme

**Figure 4:**
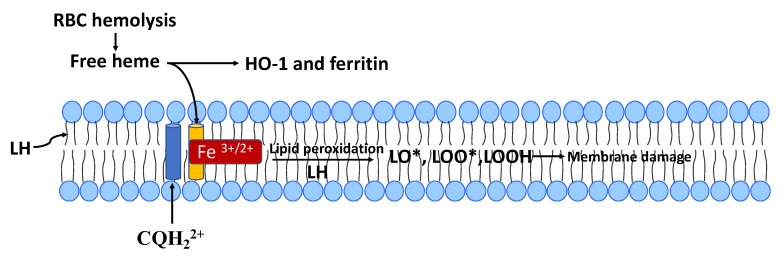
Chloroquine (CQH_2_^2+^, blue cylinder)) form a membrane-bound complex with heme (orange cylinder with Fe^3+/2+^) that promotes the peroxidation of phospholipids with polyunsaturated fatty acids (PUFAs or LH). Reactive lipid species (LO•, LOO• and LOOH) from lipid peroxidation can damage both biomembranes and membrane-bound proteins. Heme, in the absence of CQ can also cause lipid peroxidation, but this process is markedly amplified by formation of the CQ-heme complex (see text). RBC hemolysis and/or dysfunction of mitochondrial heme metabolism can produce free heme. If not metabolized by hemeoxygenase-1 (HO-1), and the released iron sequestered by ferritin, lipophilic heme can bind to biomembranes.

Although very limited, a few in vitro studies on the molecular mechanism(s) of CQ/HCQ induced oxidative stress are very relevant to COVID-19. In general, bacterial or viral sepsis and ARDS are often accompanied by the release of heme (iron-protoporphyrin I×) from RBCs and hemeproteins [108]. Heme (see Figure 1 and Figure 4) is very hydrophobic and intercalates in biomembranes, bringing redox-active iron ions in close contact with the unsaturated fatty acids of membrane phospholipids, thereby promoting lipid peroxidation [109]. Heme, in an in vitro model system forms a complex with CQ that induces membrane lipid peroxidation about five-fold higher than observed with heme alone [99,110].

Phospholipids with docosahexaenoic acid (22:6n3) were found to be particularly susceptible to the heme-CQ induced lipid peroxidation. All-racemic alpha-tocopherol, a synthetic form of vitamin E, was found to inhibit this heme-CQ-induced lipid peroxidation [99].

The retina has the highest concentration of 22:6n3, thereby contributing to the high sensitivity of the retina to oxidative stress damage [105]. The retina could, therefore, be particularly susceptible to oxidative stress damage in CQ/HCQ-treated COVID-19 patients with excess free-heme and low vitamin E levels. Heme is synthesized in mitochondria, and dysfunctional heme metabolism can result in mitochondrial oxidative stress [111]. In vitro experiments show that free-heme can cause mitochondrial dysfunction by reactive lipid species arising from heme-induced lipid peroxidation (see Figure 4). CQ/HCQ might amplify heme-induced lipid peroxidation damage to mitochondrial membranes, but this has not been investigated.

Considerable data support the view that heme, even in the absence of CQ, can promote lipid peroxidation and contribute to inflammation, cytokine production, and vascular injury [112,113]. Sparkenbaugh et al. [114] found that heme induces a systemic activation of coagulation in a mouse model. Coagulation disorders are increasingly recognized as contributors to severe COVID-19 pathology, as evidenced by increased levels of D-dimer and fibrin/fibrinogen-degradation products [115,116].

Since heme contributes to the pathology of COVID-19, heme oxygenase (HO-1) is likely to be protective against heme-induced lipid peroxidation by keeping redox-active iron out of biomembranes. HO-1 functions as an antioxidant enzyme by converting heme into biliverdin/bilirubin, ferrous ions, and carbon monoxide (CO). As indicated in Figure 4, the free iron released by HO-1 is sequestered by ferritin and blocked from ROS-generating Fenton reactions [117]. As pointed out by Hooper [118], HO-1 tissue levels tend to be low in the elderly, who are very susceptible to COVID-19 mortality. Hooper [118] also suggests that HO-1 inducers such as curcumin, resveratrol, and melatonin be investigated as potential COVID-19 treatments. In vitro studies show that resveratrol and melatonin act synergistically to induce HO-1 [119].

(13) $$\mathrm{heme}\;\overset{\mathrm{HO}-1}{\to }\;\mathrm{biliverdin}/\mathrm{bilirubin}\;+\;{\mathrm{Fe}}^{2+}\;+\;\mathrm{CO}$$

## 12. Zinc and CQ/HCQ Treatment for COVID-19

The potential use of zinc combined with HCQ has received considerable attention as a possible treatment for COVID-19, primarily based on data from a few in vitro model systems [120]. One study found that Zn^2+^, in combination with a zinc ionophore, inhibits the RNA-dependent RNA polymerase of SARS-CoV-1 and thereby inhibits viral replication in an in vitro cell culture model [121]. Significantly, this inhibition of SARS-CoV-1 replication could be achieved at a low Zn^2+^ concentration (2 µM) potentially attainable with pharmacological dosing. A second in vitro study suggested that CQ could specifically function as a zinc ionophore in an ovarian cancer cell line (A2780) and [122]. Using FluoZin-3, the study found that CQ in the presence of Zn^2+^ increased cellular uptake of Zn^2+^ in a concentration-dependent manner, resulting in apoptosis of the A2780 cells by an “enhanced” CQ toxicity. While this study suggests that CQ may function as a zinc ionophore, there are a few significant limitations. The A2780 cells were incubated with CQ for 1 h, during which some CQ could have converted to metabolites that might function as a zinc ionophore(s).

Moreover, CQ could have induced the formation of membrane-bound lipid peroxidation products with the ability to compromise the impermeability of the A2780 cells’ biomembranes to cations [123]. Alternatively, CQ could have altered the functioning of membrane proteins to promote Zn^2+^ membrane permeability. There is nothing chemically obvious about the organic structure of CQ suggesting a similarity to other Zn ionophores: if anything, CQ, as a cation, should repel metal ion cations. To prove that CQ is a Zn ionophore would require kinetic studies in a metabolically inert model system such as liposomes. Work by Dabbagh-Bazarbachi [124] et al. used this approach to establish that quercetin functions as a true Zn ionophore. If zinc combined with CQ/HCQ can clinically prevent replication of SARS-CoV-2 by inhibition of RNA-dependent RNA polymerase, such treatment would likely be most useful prophylactically or in the early phase of rapid viral replication and less useful in the late “cytokine storm”, where host-damaging inflammatory responses give rise to severe pathophysiology. A randomized, open-label trial to assess the safety and efficacy of HCQ and Zn is now underway [125].

## 13. Conclusions

**Figure 5:**
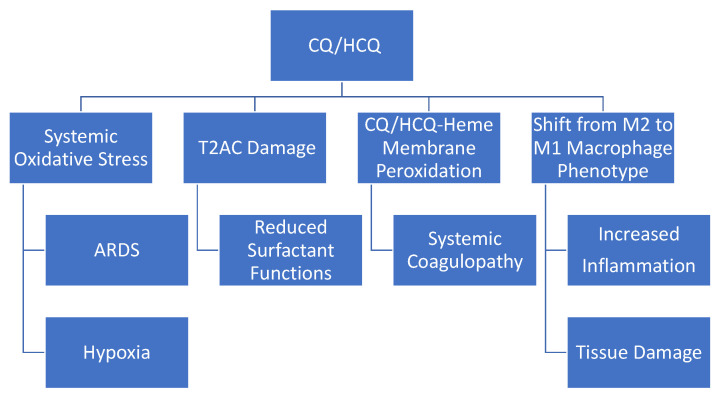
Potential adverse effects of CQ/HCQ on COVID-19 patients. CQ/HCQ-induced systemic oxidative stress could contribute to the pathology of acute respiratory distress syndrome (ARDS) and hypoxia. Hypoxia, in turn, can contribute to increased production of reactive oxygen species (ROS) from mitochondria (see text). CQ/HCQ-induced damage to type II alveolar cells (T2ACs) and surfactant function could also contribute to ARDS and hypoxia/hypoxemia. As indicated in Figure 4, CQ can form a complex with free-heme that promotes biomembrane lipid peroxidation and this process could contribute to the systemic coagulopathy found in severe COVID-19 (see text). CQ/HCQ could also alter the immune responsive by promoting a shift in macrophage phenotype from M2 to M1 (see text). The M1 macrophage phenotype is proinflammatory and promotes tissue damage. The potential adverse effects of CQ/HCQ summarized in this figure need to be further confirmed by additional in vivo and in vitro studies.

In an era when precision medicine is an ever-achievable goal, the CQ/HCQ antimalarial drugs are characterized by nonspecifically influencing many fundamental and diverse cellular structures and processes, e.g., biomembrane structure and functions, mitochondrial respiration, proton fluxes, and ROS production. The potential adverse effects of CQ/HCQ on COVID-19 patients, discussed in this review, are summarized in Figure 5. The literature reviewed here supports the overall view that CQ/HQ treatment during the hyperinflammatory phase of COVID-19 could be particularly counterproductive. In addition to affecting macrophages, as indicted in Figure 5, it is likely that CQ/HCQ indirectly influence T-cell-mediated immune response by modulating the production of ROS. ROS are known to play a direct role in modulating T-cell proliferation, differentiation, and T-cell apoptosis [126].

In vitro experiments that either support or refute the use of CQ/HCQ for COVID-19 can be misleading. Such investigations must be viewed with caution until high-quality clinical trials are completed. For example, initial in vitro work found both CQ and HCQ to be effective at inhibiting SARS-CoV-2 infection of African green monkey kidneyVeroE6 cells [127]. Subsequent work found that CQ did not inhibit SARS-CoV-2 infection of a human lung cell line (Calu-3) [128]. Human lung cells are a much more relevant cell line than African green monkey kidney cells. To date, two randomized, double-blind placebo-controlled trials testing the therapeutic potential of HCQ for COVID-19 have been reported [129,130]. HCQ was not effective in either of these trials. The clinical trial by Skipper et al. was targeted at early COVID-19 disease and also found (post hoc analysis) that self-reported use of zinc and vitamin C, along with HCQ, did not improve symptoms [129]. Moreover, adverse effects occurred more often (*p* < 0.001) in participants receiving HCQ compared to placebo [129]. A third multicenter, randomized, open-label trial found that HCQ with or without azithromycin was also not effective [131].

The potential role of ROS in COVID-19 remains to be fully elucidated. In the case of influenza A viruses, inhibition of ROS production can reduce inflammation, as measured by a decrease in virally induced cytokine production [15]. The measurement of oxidative stress parameters in CQ/HCQ COVID-19 trials would be of clinical value. Quantification of plasma F2-isoprostanes, specifically 8-epi-prostaglandin F2α, is a clinically useful and sensitive assay for assessing in vivo lipid peroxidation and oxidative stress [132]. Similarly, the plasma ratio of α-tocopherol quinine to α-tocopherol is an excellent indicator of antioxidant status [132]. Marcello et al. recently found that two oxysterols, 7-ketocholesterol and 7-beta-hydroxycholesterol, were increased in the serum COVID-19 patients, particularly those with severe signs. These two oxysterols are also useful biomarkers for in vivo lipid peroxidation [133].
